# Maternal Cigarette Smoking and Congenital Upper and Lower Limb Differences: A Systematic Review and Meta-Analysis

**DOI:** 10.3390/jcm12134181

**Published:** 2023-06-21

**Authors:** Jevan Cevik, Omar Salehi, James Gaston, Warren M. Rozen

**Affiliations:** 1 Department of Plastic and Reconstructive Surgery, Peninsula Health, Melbourne, VIC 3199, Australia; 2 Peninsula Clinical School, Central Clinical School, Faculty of Medicine, Monash University, Melbourne, VIC 3199, Australia

**Keywords:** maternal smoking, congenital birth defects, congenital limb differences, polydactyly, syndactyly

## Abstract

Maternal smoking during pregnancy has been associated with adverse effects on foetal development, including congenital limb anomalies. This systematic review aimed to provide an updated assessment of the association between maternal smoking during pregnancy and the risk of congenital limb anomalies. A systematic search was conducted to identify relevant studies published up to February 2023. Studies reporting on the relationship between maternal smoking during pregnancy and congenital digital anomalies or congenital limb reduction defects were included. Two independent reviewers screened the studies, extracted data, and assessed the quality of the included studies. Meta-analyses were performed to estimate the pooled odds ratios with 95% confidence intervals using fixed and random-effects models. In total, 37 publications comprising 11 cohort and 26 case-control studies were included in the systematic review. The meta-analysis demonstrated a significant increased risk of congenital limb reduction defects (pooled OR: 1.27, 95% CI: 1.18–1.38) in infants born to mothers who smoked during pregnancy. Similarly, a significant relationship was observed for the development of polydactyly/syndactyly/adactyly when considered as a single group (pooled OR: 1.32, 95% CI: 1.25–1.40). Yet, in contrast, no significant association was observed when polydactyly (pooled OR: 1.06, 95% CI: 0.88–1.27) or syndactyly (pooled OR: 0.91, 95% CI: 0.77–1.08) were considered individually. This systematic review provides updated evidence of a significant relationship between maternal smoking during pregnancy and increased risk of congenital limb anomalies. These findings highlight the potential detrimental effects of smoking on foetal limb development and underscore the importance of smoking cessation interventions for pregnant women to mitigate these risks.

## 1. Introduction

Congenital limb defects refer to structural abnormalities that occur during foetal development and affect the limbs of newborns. These abnormalities can vary in severity, ranging from minor deformities like extra fingers or toes, to more severe malformations like complete absence of a limb. Despite being relatively rare, with an estimated prevalence of 5–27 per 10,000 live births [1,2,3,4,5,6,7,8], these anomalies can have significant impacts on the affected children and their families, including functional, cosmetic, and psychological implications [9,10,11]. There remains limited knowledge about the risk factors associated with giving birth to children with congenital limb defects, and, in most cases, these defects arise spontaneously without a clearly identifiable cause.

It has long been demonstrated that environmental exposure of the mother to various aetiological agents during pregnancy can result in significant birth defects, including limb anomalies. Among these include teratogenic medications such as thalidomide or anticonvulsants and many others [12,13,14,15,16,17,18,19]. Among the environmental factors, maternal exposure to cigarette smoke during pregnancy has been previously shown to be a significant risk factor for congenital birth defects [20,21]. Cigarette smoke is a complex mixture of over 7000 chemicals, including nicotine, carbon monoxide, and various other toxic agents, many of which can cross the placenta and potentially affect foetal development [22,23,24]. Maternal smoking during pregnancy is a well-known risk factor for various adverse health outcomes, such as low birth weight, preterm birth, and perinatal death [25,26,27,28]. Given this, the association between maternal smoking and congenital defects such as congenital limb anomalies has been an area of interest among epidemiologists and surgeons treating these conditions, however, the relationship between these two variables remains controversial. Multiple previous observational studies have revealed conflicting findings, with some showing positive associations between maternal smoking and congenital limb differences [29,30,31,32,33] and others revealing no significant association [34,35,36,37,38]. 

A previous systematic review published by Hackshaw et al. in 2011 suggested an association between maternal cigarette smoking during pregnancy and the risk of congenital limb defects in children [20]. For the subsection of their review focusing on congenital limb defects, they included eight studies assessing the impact of maternal smoking on congenital limb reduction defects and six on congenital digital anomalies. The obtained effect sizes were 1.26 (95% CI: 1.15–1.39) for congenital limb reduction defects and 1.18 (95% CI: 0.99–1.41) for congenital digital anomalies. However, since the publication of that review, numerous additional studies have been published, which need further consideration. Moreover, in this previous study, because their objective was stated to be inclusive and objective, no rigorous quality assessment was utilized to select studies for inclusion into their meta-analysis, leaving the results subject to increased bias. The existing literature on this topic has multiple limitations such as small sample sizes, methodological variations, and potential confounding factors, which may impact the validity of the findings. As a result, the aim of this study is to perform an updated systematic review to capture recent publications in this field, thoroughly assess the quality of the included studies, and perform a revised and reliable meta-analysis. This updated review will critically evaluate the current evidence and provide a synthesis of the relationship between maternal smoking during pregnancy and the risk of congenital limb defects in children.

## 2. Methods

### 2.1. Study Identification

**Figure 1:**
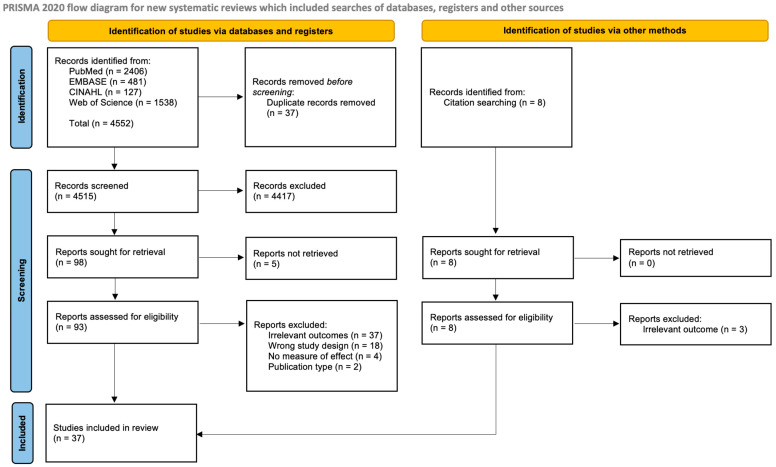
PRISMA flowchart of study selection [39].

This systematic review adhered to the Preferred Reporting in Systematic Review and Meta-Analysis (PRISMA) guidelines (Figure 1) [39]. A comprehensive and systematic search was conducted to identify relevant studies examining the association between maternal smoking during pregnancy and congenital limb defects in children. Multiple electronic databases including PubMed, Embase, CINAHL, and Web of Science were thoroughly searched from their inception up to the 6th of February 2023. The search strategy utilized a combination of relevant keywords and MeSH terms to ensure a comprehensive coverage of the literature. The following search terms were used: (“Maternal Smoking” OR “Prenatal Smoking” OR “Periconceptional smoking” OR “Gestational smoking”) AND (“Birth defects” OR “Congenital anomalies” OR “Congenital abnormalities” OR “Congenital defects” OR “Congenital digital anomalies” OR “Polydactyly” OR “Syndactyly” OR “Adactyly” OR “Limb Reduction”). The search was not limited by language or publication status to minimize the risk of language bias and publication bias. In addition to the electronic database searches, the reference lists of all included studies and relevant reviews were thoroughly screened for any additional studies that may have been missed in the initial search. 

### 2.2. Study Inclusion and Data Extraction

Studies were included in the systematic review if they met the following predefined criteria: Firstly, study designs were limited to original randomised control, case-control, or cohort studies that evaluated the association between maternal smoking during pregnancy and congenital limb defects in children. The included outcomes were restricted to two main subgroups: congenital digital anomalies (comprising polydactyly, syndactyly, or adactyly) and congenital limb reduction defects. These studies could be conducted retrospectively or prospectively, allowing for a wide range of study designs to be included in the analysis. Secondly, only studies that provided sufficient data to calculate odds ratios (ORs) or relative risks (RRs) with 95% confidence intervals (CIs), or provided these statistics in the study, were eligible for consideration of quantitative analysis. Studies reporting outcomes which had insufficient information to obtain quantitative data were included in the presented tables and study but were unable to be considered for meta-analysis. Lastly, this review was limited to studies with human subjects, as the review focused on the impact of maternal smoking during pregnancy on congenital limb defects in human children.

On the other hand, studies were excluded from the systematic review if they were case reports, reviews, conference presentations, cross-sectional studies, editorials, letters to the editor, animal studies, or if they did not report relevant outcomes. These exclusion criteria were applied to ensure that only original research studies with relevant and reliable data were included in the analysis, while excluding studies that were not primary research or lacked relevant outcome data. 

The inclusion and exclusion criteria were applied in a systematic and rigorous manner to select studies that were most relevant to the research question and ensure the robustness of the systematic review. Titles and abstracts of all identified articles in the search were reviewed by two independent reviewers (JC/OS) who conducted the study selection process, and any discrepancies were resolved through discussion and consensus or the involvement of a third reviewer where necessary (JG). 

Data were extracted into data extraction tables into a shared spreadsheet in Microsoft Excel. A comprehensive and systematic approach was employed to gather relevant information from each included study. Multiple data points were carefully extracted to provide a comprehensive overview of the included studies. These data points included the title, authors, publication year, country of the study population, study design, study period, data source, details of exposed group and controls, period of smoking, cases of congenital limb defects among each group, confounding factors adjusted for, and study outcomes measured. Smoking status was considered as a binary variable, where studies had stratified patients by numbers of cigarettes per day; where possible, numbers were totalled to compare smokers and non-smokers dichotomously. Similarly, for congenital digital anomalies, the presence of any of polydactyly, syndactyly, or adactyly was considered as a case given that these were the consistent variables reported amongst included studies. The presence of other digital anomalies such as clinodactyly, brachydactyly, macrodactyly, and others were not reported amongst any found study and therefore were not assessed. 

### 2.3. Quality Assessment

The quality of the included studies was assessed using the Newcastle–Ottawa Scale (NOS) for case-control and cohort studies [40]. The NOS is a widely used tool for assessing the methodological quality of non-randomized studies. It consists of three domains: selection of study groups, comparability of study groups, and assessment of outcome for cohort studies or exposure for case-control studies. Each domain is assessed based on a set of criteria, and the total score indicates the overall quality of the study. The NOS assigns stars to each criterion, with a higher number of stars indicating higher quality or a lower risk of bias. Quality assessment was performed independently by two reviewers (JC/OS), and any discrepancies were resolved through discussion and the involvement of a third reviewer if necessary (JG). 

When assessing the comparability of each study, stars were awarded to studies if they had adjusted their analyses for one or more of maternal age/paternal age, maternal diabetes, maternal obesity, family history of limb anomaly, and maternal alcohol consumption. These confounders were chosen as previous studies have displayed evidence that these factors may potentially influence the formation of congenital limb anomalies in children [41,42,43,44,45]. It is not currently known which of these factors confers the highest risk, and therefore stars were awarded for any of the five confounders that were adjusted for in the included studies. A single star was awarded for each of the aforementioned confounding factors adjusted for, up to a maximum of two stars.

### 2.4. Statistical Analysis

A meta-analysis was conducted to estimate the overall effect size of the association between maternal smoking during pregnancy and congenital limb defects in children. Heterogeneity was assessed using the I^2^ statistic and pooled odds ratios with 95% confidence intervals were calculated using fixed-effects models when heterogeneity was low (I^2^ < 50%) or random-effects models when heterogeneity across studies was high. Meta-analyses were performed separately for the two subgroups of congenital digital anomalies and congenital limb reduction defects. Studies were deemed of sufficient quality to be included in the meta-analysis if they were assessed to be of “fair” or “good” quality as determined using the Newcastle–Ottawa Scale. Publication bias was assessed using funnel plots and visually inspected for asymmetry. All statistical analyses were conducted using statistical software using the Review Manager program [46].

## 3. Results

A total of 4552 (2406 from PubMed + 481 from EMBASE + 127 from CINAHL + 1538 from Web of Science) studies were initially identified through a comprehensive search of electronic databases and additional sources. After removing duplicates, 4515 studies were eligible for initial review. Following screening of study titles and abstracts, 106 studies were selected for full-text review. Five studies did not have available full texts. Following the full-text review, 37 publications were finally included in this systematic review based on the predefined inclusion and exclusion criteria (Figure 1).

The included studies were published between 1978 and 2022 and were conducted in various countries, including the United States, Sweden, Denmark, China, Japan, Finland, Hungary, Norway, Poland, and Australia. In total, 38 studies from 37 publications, comprising 11 cohort and 27 case-control studies, investigated the association between maternal cigarette smoking during pregnancy and the risk of congenital limb anomalies (one publication included descriptions of two case-control studies from different populations and methodologies [47]). This included 12 studies which included outcomes of congenital digital anomalies (polydactyly, syndactyly, or adactyly) and 26 studies which included outcomes on congenital limb reduction defects. In total, there were 26,787 identified cases of congenital limb anomalies comprising 16,330 cases of congenital digital anomalies and 10,457 cases of congenital limb reduction defects. Table 1 and Table 2 summarise the characteristics of the included studies.

### 3.1. Quality Assessment of Included Studies

The quality of the included studies was assessed using the Newcastle–Ottawa Scale for cohort and case-control studies. In total, six studies were considered of “good” quality, 14 of “fair” quality, and 18 of “poor” quality (Table 3). Most studies were deemed of poor quality due to a lack of adjustment of effect measures for confounding factors (maternal/paternal age, maternal diabetes, maternal obesity, family history of limb anomaly, and maternal alcohol consumption). This adjustment was considered only in the context of congenital limb defects and not other reported outcomes. Poor quality studies were excluded from the meta-analysis. 

**Figure 2:**
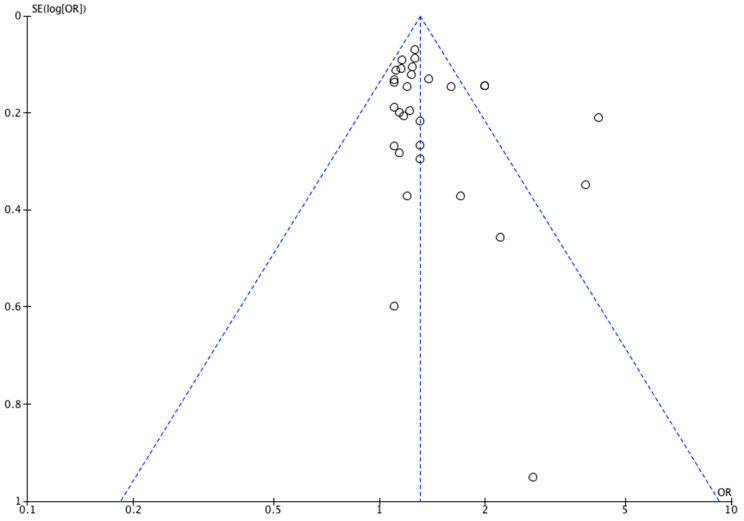
Funnel plot of included studies reporting on congenital limb reduction defects.

The funnel plot (Figure 2) for congenital limb reduction defects was mostly symmetrical, however, it showed an asymmetry in the lower left corner, suggesting a lack of studies that demonstrated the protective effects of maternal smoking against defects in children, which may suggest potential publication bias towards studies with larger sample sizes. Given that quantitative data was available for less than 10 studies reporting on each outcome of congenital digital anomalies, forest plots were not performed. 

### 3.2. Meta-Analyses

As previously mentioned, meta-analyses in this study were performed only on studies that were deemed of good or fair quality as assessed by the Newcastle–Ottawa Scale.

### 3.3. Congenital Digital Anomalies

In total, 12 studies provided data on the association between maternal cigarette smoking during pregnancy and congenital digital anomalies, including polydactyly, syndactyly, and adactyly. Nine were of sufficient quality to be included in the quantitative analysis. A meta-analysis was conducted to estimate the pooled odds ratio (OR) and 95% confidence interval (CI) for the association between maternal cigarette smoking during pregnancy and the risk of congenital digital anomalies.

**Figure 3:**
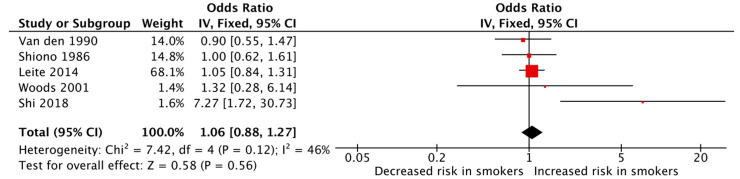
Forest plot displaying the measures of effects for studies reporting polydactyly as the outcome [36,37,50,51,52].

**Figure 4:**
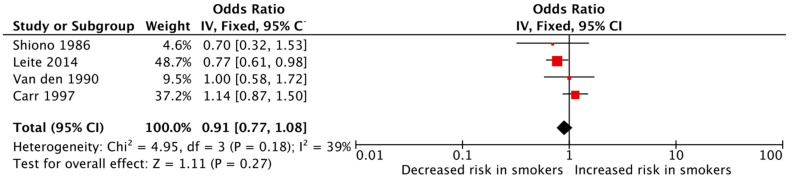
Forest plot displaying the measures of effects for studies reporting syndactyly as the outcome [36,37,49,51].

The meta-analyses of the various outcomes showed no significant association between maternal cigarette smoking during pregnancy and an increased risk of polydactyly (pooled OR: 1.06, 95% CI: 0.88–1.27, Figure 3) or syndactyly (pooled OR: 0.91, 95% CI: 0.77–1.08, Figure 4) individually. The heterogeneity among the studies was low for both meta-analyses with an I^2^ of 46% and 39%, respectively, for polydactyly and syndactyly. 

**Figure 5:**
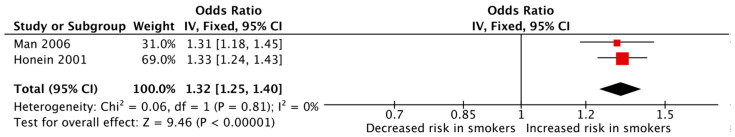
Forest plot displaying the measures of effects for studies reporting polydactyly/syndactyly/adactyly as the outcome [29,30].

In contrast, the meta-analysis of studies that reported the outcome of polydactyly/syndactyly/adactyly was found to be significant, indicating an increased risk of children developing polydactyly or syndactyly or adactyly among mothers who smoked during pregnancy (pooled OR: 1.32, 95% CI: 1.25–1.40, Figure 5). Heterogeneity was low (I^2^ = 0%). However, this analysis was limited by the fact that it only included two studies.

### 3.4. Congenital Limb Reduction Defects

**Figure 6:**
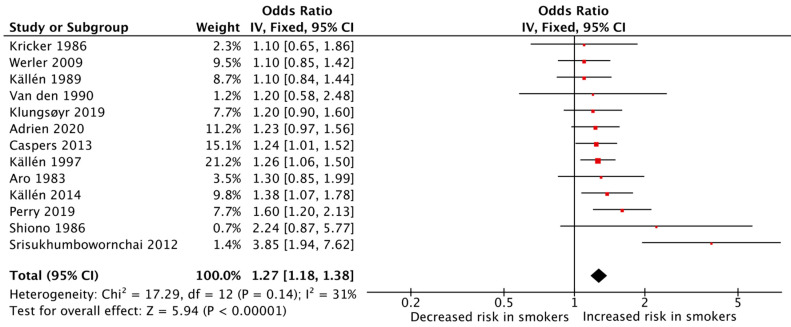
Forest plot for displaying the measures of effects for studies reporting on congenital limb reduction defects [31,33,34,35,36,37,38,43,55,58,66,69,70].

In total, 31 studies provided data on the association between maternal cigarette smoking during pregnancy and congenital limb reduction defects, and 13 were of sufficient quality for quantitative analysis. The meta-analysis of these studies showed that maternal cigarette smoking during pregnancy was significantly associated with an increased risk of congenital limb reduction defects (pooled OR: 1.27, 95% CI: 1.18–1.38, Figure 6). The heterogeneity among the studies was low (I^2^ = 31%).

## 4. Discussion

Despite the well-known risks, smoking remains a significant global health burden and is a leading cause of preventable deaths worldwide, resulting in an estimated 8 million deaths each year [73]. Furthermore, maternal cigarette smoking during pregnancy has been consistently shown to be a significant risk factor for adverse pregnancy outcomes and congenital birth defects. Many studies have investigated the risk of congenital birth defects among mothers who smoke during pregnancy such as congenital limb defects, congenital heart defects, neural tube defects, urogenital defects, cleft lip/palate, and others [20,21]. Herein, we have systematically reviewed the literature assessing the impact of maternal cigarette smoking during pregnancy on congenital limb defects including congenital digital anomalies and congenital limb reduction defects.

The findings of the meta-analyses in this study highlight a significant increased risk of congenital limb reduction defects (pooled OR: 1.27, 95% CI: 1.18–1.38) among offspring of smoking mothers compared to non-smoking counterparts. In contrast, the results for individual congenital digital anomalies were not significant, with meta-analyses of studies reporting outcomes of polydactyly and syndactyly individually not being found to show any significant association (polydactyly pooled OR: 1.06, 95% CI: 0.88–1.27, syndactyly pooled OR: 0.91, 95% CI: 0.77–1.08). Yet, when studies that reported the outcome of any one of polydactyly/syndactyly/adactyly were meta-analysed, a significantly increased risk of congenital digital anomalies among smokers was observed (pooled OR: 1.32, 95% CI: 1.25–1.40). However, these results must be interpreted with caution as only two studies were available for quantitative analysis for the latter meta-analysis. Overall, there seems to be an association between maternal smoking and the development of limb reduction defects among offspring; however, the results are less clear for congenital digital anomalies. It is unclear why this disparity exists, perhaps the relative paucity of the literature pertaining to congenital digital anomalies has limited the ability of this review to find a clear association if one does in fact exist.

These results are consistent with a previous meta-analysis conducted over a decade ago, which also reported a similar positive association between maternal smoking during pregnancy and the risk of congenital digital anomalies and limb reduction defects [20]. This previous review by Hackshaw et al. included eight studies assessing the impact of maternal smoking on congenital limb reduction defects and six on congenital digital anomalies comprising a total of 11 studies. The obtained effect sizes were 1.26 (95% CI: 1.15–1.39) for congenital limb reduction defects and 1.18 (95% CI: 0.99–1.41). These results are similar to the effect sizes obtained from our present review, despite finding 26 additional publications and employing a rigorous quality assessment of the included studies. By including a larger number of studies, the meta-analyses in this study provide a more comprehensive and up-to-date assessment of the association between smoking during pregnancy and congenital digital anomalies and limb reduction defects. The rigorous quality assessment of the included studies also ensured that only studies with robust methodologies and reliable data were included in the meta-analyses, enhancing the credibility of the findings.

Smoking during pregnancy has been shown to increase the risk of birth defects through various mechanisms. The toxic chemicals in tobacco smoke, such as nicotine and carbon monoxide, can cross the placenta and directly damage developing foetal tissues. Nicotine, a vasoactive agent, can also constrict blood vessels, reduce blood flow, and impair oxygen delivery to the foetus, causing disruption in development and growth [74]. Furthermore, carbon monoxide in tobacco smoke binds to haemoglobin in the blood, reducing its ability to carry oxygen, further enhancing oxygen deprivation in the developing foetus. This is thought to cause chronic hypoxia resulting in a reflex whereby blood is shunted away from the limbs (particularly the lower limbs) and preferentially redirected towards the foetal heart and brain [75]. This would inevitably cause a lack of vital oxygen and nutrients being delivered to the foetal limbs. Additionally, smoking during pregnancy can result in oxidative stress, which results in an imbalance between the production of harmful free radicals and the body’s ability to neutralise them [76,77]. This oxidative stress can damage cellular DNA and disrupt cellular processes critical for foetal development [76,77]. Moreover, tobacco smoke contains toxins such as polycyclic aromatic hydrocarbons that can also interfere with cellular processes involved in DNA synthesis, cell proliferation, and differentiation [78]. Numerous other mechanisms have been proposed, including epigenetic changes and vascular endothelial damage [79,80,81]. Overall, the mechanisms by which smoking causes birth defects are complex and multifactorial, involving a combination of direct and indirect toxic effects.

In addition to lifestyle factors such as maternal cigarette smoking or alcohol consumption, numerous other risk factors for congenital limb differences among offspring exist. Genes play a crucial role in the development and patterning of limbs during embryonic development. Mutations or alterations in specific genes can disrupt the normal limb development process, leading to congenital limb anomalies. For example, mutations in genes such as the HOX gene family, SHH gene, and TBX genes (among many others) have been associated with limb development abnormalities [82]. Furthermore, numerous genetic syndromes are connected to congenital limb differences such as Fanconi Anaemia, Roberts Syndrome, and Holt-Oram Syndrome [82,83]. Also, environmental exposure of the mother to toxins, radiation, pesticides, or other harmful substances has also been linked with the development of congenital limb difference [84,85,86]. 

This systematic review has several strengths. Firstly, this review employed a broad and comprehensive search strategy to identify relevant studies, which can help minimize the risk of missing important evidence. Additionally, the study selection process was rigorous, involving careful screening of studies based on predefined inclusion and exclusion criteria. Furthermore, the review included a methodical assessment of study quality, ensuring that only studies of sufficiently high quality were included in the analysis.

However, there are several limitations to consider. One limitation is the lack of high-quality studies assessing the relationship between cigarette smoking and congenital digital anomalies. While we found 12 studies assessing this relationship, the various outcomes reported in each study differ, making meaningful meta-analysis of these outcomes difficult. Hence, the relationship observed between maternal smoking and the development of polydactyly/syndactyly/adactyly was based on only two studies in the meta-analysis, making this relationship less reliable. Another limitation is the potential for recall bias in case-control studies that used interviews as a method of investigation. Interviews rely on self-reporting by participants, which can be subject to recall bias and may not always accurately capture exposure information, such as smoking during pregnancy. Additionally, despite efforts to only include studies in the meta-analysis that had adjusted for confounding variables, residual confounding may still be present in the included studies. For example, while the review mentioned adjustment for significant confounding variables such as maternal age, diabetes, obesity, family history of limb anomaly, and alcohol consumption, there may be other unmeasured or unknown confounding factors that could impact the relationship between maternal smoking and congenital limb anomalies. A study protocol was not registered prior to the conduct of this study. Furthermore, publication bias may be a limitation, as indicated by the asymmetric funnel plot in congenital limb reduction defects. Additionally, unfortunately we were unable to assess a dose–response relationship between studies due to inconsistent reporting of number of cigarettes smoked by mothers in the included studies. Lastly, there was no consideration of the effect of passive smoking or second-hand smoke exposure in the meta-analysis. Maternal exposure to second-hand smoke during pregnancy has been shown to be connected with congenital defects and limb deficiencies, and could potentially confound the relationship between maternal smoking and congenital limb anomalies as the non-smoking mothers may experience similar detrimental effects of cigarette smoke from second-hand smoking [31,57,87]. 

## 5. Conclusions

This systematic review provides updated evidence of the relationship between maternal cigarette smoking during pregnancy and congenital limb anomalies, specifically congenital digital anomalies and congenital limb reduction defects. The detrimental effects of smoking on foetal development are well-established, and our review further reinforces the harmful impact of maternal smoking on limb development. Further research is warranted to better understand the underlying mechanisms linking maternal smoking and congenital limb anomalies, particularly congenital digital anomalies, and to explore potential strategies to mitigate these risks. This review underscores the critical need for effective smoking cessation interventions for pregnant women to ensure optimal foetal development.

## Figures and Tables

**Table 1:** Characteristics of included studies assessing impact of maternal smoking on digital anomalies.

Author	Year	Country	Study Design	Study Period	Data Source	Control Group	Period of Smoking	Smokers		Non-Smokers		OR/RR (95% CI) *	Potential Confounders Adjusted	Outcomes Measured
								Cases	Controls	Cases	Controls			
Kelsey et al. [48]	1978	USA	Case-Control	1974–1976	Birth data from five Connecticut hospitals	Births without congenital defects	T1	NS (total 50)	980	NS (total 50)	1988	NS	-	Polysyndactyly
Shiono et al. [36]	1986	USA	Cohort	1974–1977	Kaiser Permanente Birth Defects Study	Births without congenital defects	T1	27	NS	80	NS	P: 1.0 (0.6–1.6) S: 0.7 (0.3–1.5)	Maternal age Ethnicity Alcohol use	Polydactyly and Syndactyly separately
Van Den Eeden et al. [37]	1990	USA	Case-Control	1984–1986	Washington State Birth Records	Births without congenital defects	NS	NS (183 total)	1037	NS (183 total)	3286	P: 0.9 (0.6–1.5) S: 1.0 (0.6–1.7) A: 0.9 (0.3–2.9)	Maternal age Parity	Polydactyly, Syndactyly and Adactyly separately
Carr [49]	1997	USA	Case-Control	1987–1995	Washington State Birth Events Records Data Base	Births without congenital limb defects	T1-T3	289	1186	1066	4990	P: 1.05 (0.85–1.30) S: 1.14 (0.87–1.50)	Gravidity/Parity Marital status Paternal age ** Race	Polydactyly and Syndactyly separately
Kallen (C) [32]	2000	Sweden	Cohort	1983–1996	The Swedish Registry of Congenital Malformations and the National Board of Health Medical Birth Registry	Births without congenital defects	T1	758	347, 513	2392	1, 066, 298	P: 1.09 (0.98–1.22) S: 0.85 (0.76–0.96)	-	Polydactyly and Syndactyly separately
Honein et al. [29]	2001	USA	Case-Control	1997–1998	National Vital Statistics (USA)	Births without congenital defects	NS	NS (5573 total)	NS (6, 134, 773 total)	NS (5573 total)	NS (6, 134, 773 total)	1.33 (1.23–1.43)	Maternal age Maternal race Education	Polydactyly or Syndactyly or Adactyly together
Woods and Raju [50]	2001	USA	Cohort	1998–1999	TriHealth Hospital System	Births without congenital defects	NS	2	1687	12	14, 263	1.32 (0.28–6.12)	Maternal age Race Maternal diabetes	Polydactyly
Man and Chang [30]	2006	USA	Case-Control	2001–2002	U.S. Natality Database	Births without congenital defects	NS	805	4366	1280	9062	1.31 (1.18–1.45)	Marital status Maternal diseases Maternal diabetes Maternal Hypertension Previous premature delivery Maternal chronic disease Rh sensitivity	Polydactyly, Syndactyly or Adactyly
Leite et al. [51]	2014	Denmark	Cohort	1997–2010	Danish Medical Birth Register	Births without congenital defects	T1-T3	189	147, 218	941	641, 356	P: 1.05 (0.84–1.32) S: 0.77 (0.61–0.98)	Maternal age Year of birth Maternal marital status	Polydactyly and Syndactyly separately
Kallen (D) [34]	2014	Sweden	Cohort	1998–2010	The Swedish Registry of Congenital Malformations and the National Board of Health Medical Birth Registry	Births without congenital defects	NS	195	NS	2137	NS	0.88 (0.76–1.02)	Year of birth Maternal age Parity BMI	Polydactyly or Syndactyly together
Shi et al. [52]	2018	China	Case-Control	2015–2017	Tongji Hospital Department of Orthopaedic or Paediatric surgery	Births without polydactyly	NS	9	4	134	282	7.27 (1.72–30.72)	Family income Household registration Family history of polydactyly Close relative marriage Maternal depression during pregnancy Education level Newborn sex	Polydactyly
Tsuchida et al. [53] ***	2021	Japan	Cohort	2011–2014	Japan Environment and Children’s Study	Births without congenital defects	NS	52	16, 864	156	53, 356	P (fingers): 1.12 (0.64–1.96) S (toes): 0.93 (0.47–1.82)	Maternal age Maternal BMI Maternal diabetes Marital status Education level Household income IVF or artificial insemination Alcohol intake Folic acid intake Antihypertensive/Anti-convulsant use Retinoic acid intake	Polydactyly of fingers, Polydactyly of toes, Syndactyly of fingers, Syndactyly of toes separately

* Adjusted odds ratios used where provided, otherwise crude odds rations calculated from available data. ** Not included in adjustment for polydactyly OR. *** Unable to calculate overall odds ratio as groups presented were not independent. OR = Odds ratio. RR = Relative risk. NS = not specified. P = Polydactyly. S = Syndactyly. A = Adactyly. T1 = First Trimester. T1-3 = Throughout Pregnancy.

**Table 2:** Characteristics of included studies assessing impact of maternal smoking on congenital limb reduction defects.

Author	Year	Country	Study Design	Study Period	Data Source	Control Group	Period of Smoking	Smokers		Non-Smokers		OR/RR (95% CI) *	Potential Confounders Adjusted	Outcomes Measured
								Cases	Controls	Cases	Controls			
Aro et al. (A) [43]	1983	Finland	Case-Control	1964–1977	The Finnish registry of congenital malformations	Births without limb reduction defects	NS	NS (total 453)	NS (total 964, 397)	NS (total 453)	NS (total 964, 397)	1.3 (0.9–2.0)	Maternal age Alcohol use	Limb reduction defects
Aro et al. (B) [54]	1984	Finland	Case-Control	1964–1977	The Finnish registry of congenital malformations	Births without limb reduction defects	NS	NS (total 453)	NS	NS (total 453)	NS	1.3 (0.7–2.3)	Maternal age Alcohol use Other adjustments not specified	Limb reduction defects
Shiono et al. [36]	1986	USA	Cohort	1974–1977	Kaiser Permanente Birth Defects Study	Births without congenital defects	NS	8	NS (total 28, 810)	9	NS (total 28, 810)	2.2 (0.9–5.8)	Maternal age Ethnicity Alcohol use	Limb reduction defects
Kicker et al. [35]	1986	Australia	Case-Control	1970–1981	Two Australian States	Births without congenital defects	T1	37	25	108	214	1.1 (0.7–1.9)	Maternal age Sex of infant Parity Father’s occupation “Pregnancy factors”	Limb reduction defects
Kallen (A) [55]	1989	Sweden	Cohort	1983–1986	The Swedish Registry of Congenital Malformations and the National Board of Health Medical Birth Registry	Births without limb reduction defects	NS	65	NS	141	NS	1.1 (0.8–1.4)	Maternal age Year of birth Parity	Limb reduction defects
Van Den Eeden et al. [37]	1990	USA	Case-Control	1984–1986	Washington State Birth Records	Births without congenital defects	NS	NS (total 35)	1037	NS (total 35)	3286	1.2 (0.6–2.5)	Maternal age Parity	Limb reduction defects
Czeizel et al. (A) [56]	1994	Hungary	Case-Control	1975–1984	Hungarian congenital abnormalities registry and medical clinics	Births without limb reduction defects	T1-T3	168	100	369	437	1.99 (1.50–2.64)	-	Limb reduction defects
Wasserman et al. [57]	1996	USA	Case-Control	1987–1988	California Birth Defects Monitoring Program	Births without congenital defects	T1	46	114	129	364	1.14 (0.77–1.69)	-	Limb reduction defects
Kallen (B) [58]	1997	Sweden	Cohort	1983–1993	The Swedish Registry of Congenital Malformations and the National Board of Health Medical Birth Registry	Births without congenital limb reduction defects	T1	190	299, 3715	420	814, 974	1.26 (1.06–1.50)	Maternal age Parity Maternal schooling Month/Year of birth	Limb reduction defects
Carr [49]	1997	USA	Case-Control	1987–1995	Washington State Birth Events Records Data Base	Births without congenital limb defects	T1-T3	42	1186	147	4990	1.10 (0.76–1.58)	Gravidity Marital status	Limb reduction defects
Shaw et al. [59]	1999	USA	Case-Control	1987–1988	California Birth Defects Monitoring Program	Births without congenital defects	T1	44	169	121	565	1.22 (0.83–1.79)	-	Limb reduction defects
Martinez-Frias et al. [47]	1999	Spain	Case-Control	1975–1984	Hungarian Congenital Abnormality Registry	Births without congenital defects	NS	10	3	10	17	2.72 (0.42–17.50)	Maternal age Birth weight Gestational age Pregnancy order	Poland Syndrome
Martinez-Frias et al. [47]	1999	Spain	Case-Control	1976–1997	Spanish Collaborative Study of Congenital Malformations	Births without congenital defects	NS	13	9	18	21	1.10 (0.34–3.55)	Maternal age Birth weight Gestational age Pregnancy order	Poland Syndrome
Kallen (C) [32]	2000	Sweden	Cohort	1983–1996	The Swedish Registry of Congenital Malformations and the National Board of Health Medical Birth Registry	Births without congenital defects	T1	298	347, 513	725	1066, 298	1.26 (1.10–1.44)	-	Limb reduction defects
Shaw et al. [60]	2002	USA	Case-Control	1987–1989	California Birth Defects Monitoring Program	Births without major congenital defects	T1	44	114	119	360	1.17 (0.78–1.75)	-	Limb reduction defects
Carmichael et al. (A) [61]	2004	USA	Case-Control	1987–1988	California Birth Defects Monitoring Program	Births without congenital defects	T1	28	49	64	128	1.14 (0.66–1.99)	-	Limb reduction defects
Czeizel et al. (B) [62]	2004	Hungary	Case-Control	1975–1984	Hungarian congenital abnormalities registry	Births without congenital defects	T1-T3	168	100	369	437	1.99 (1.50–2.64)	-	Limb reduction defects
Carmichael et al. (B) [63]	2006	USA	Case-Control	1987–1988	California Birth Defects Monitoring Program	Births without congenital defects	T1	28	47	17	48	1.7 (0.8–3.5)	-	Limb reduction defects
Carmichael et al. (C) [64]	2006	USA	Case-Control	1987–1988	California Birth Defects Monitoring Program	Births without major congenital defects	T1	28	101	68	330	1.3 (0.8–2.2)	-	Limb reduction defects
Robitaille et al. [65]	2009	USA	Case-Control	1997–2003	National Birth Defects Prevention Study	Births without congenital defects	T1-T3	115	998	412	3958	1.11 (0.89–1.38)	-	Limb reduction defects
Werler et al. [38]	2009	USA	Case-Control	1997–2004	National Birth Defects Prevention Study	Births without congenital defects	NS	73	1122	294	4764	1.1 (0.85–1.43)	Maternal age Education Parity Ethnicity Medication use State of maternal residence	Transverse limb reduction defects
Srisukhumbowornchai et al. [66]	2012	USA	Case-Control	2003–2007	National Birth Defects Prevention Study	Births without congenital defects	T1-T3	17	48	NS	562	3.85 (1.95–7.64)	Maternal age Race Education BMI	Limb reduction defects
Caspers et al. [31]	2013	USA	Case-Control	1997–2007	National Birth Defects Prevention Study	Births without congenital defects	T1-T3	172	1439	529	5210	1.24 (1.01–1.53)	Study site, sex, ethnicity, education, season of conception, pregnancy intention, periconceptional vasoactive medication use, folic acid use, alcohol use	Limb reduction defects
Kallen (D) [34]	2014	Sweden	Cohort	1998–2010	The Swedish Registry of Congenital Malformations and the National Board of Health Medical Birth Registry	Births without congenital defects	NS	64	NS	526	NS	1.38 (1.07–1.78)	Year of birth Maternal age Parity BMI	Limb reduction defects
Pace et al. [67]	2018	USA	Case-Control	1997–2009	National Birth Defects Prevention Study	Births without congenital defects	T1-T3	179	1685	704	7672	1.16 (0.97–1.38)	-	Limb reduction defects
Perry et al. [33]	2019	USA	Cohort	2006–2015	Ohio National Birth Records	Births without congenital defects	T1	76	247, 863	194	1, 102, 969	1.6 (1.2–2.1)	Maternal age Race Maternal diabetes mellitus Socioeconomic status Medicaid status	Limb reduction defects
Choi et al. [68]	2019	USA	Case-Control	1997–2012	National Birth Defects Prevention Study	Births without major congenital defects	NS	117	967	497	4718	1.15 (0.93–1.42)	-	Limb reduction defects
Klungsoyr et al. [69]	2019	Norway	Cohort	1999	Medical Birth Registry of Norway	Births without limb reduction defects	NS	56	134, 295	246	739, 908	1.2 (0.9–1.6)	Maternal age Year of birth Folate/vitamin use Maternal diabetes	Limb reduction defects
Adrien et al. [70]	2020	USA	Case-Control	1997–2011	National Birth Defects Prevention Study	Births without congenital defects	T1	123	2075	477	9454	1.23 (0.97–1.56)	Maternal age Race/ethnicity Education Previous live births Study centre	Transverse limb reduction defects
Materna-Kiryluk et al. [71]	2021	Poland	Case-Control	1998–2010	Polish Registry of Congenital Malformations	Births without congenital defects	T1	103	35	491	718	4.18 (2.77–6.30)	Gestational age Birth weight Gravidity	Limb reduction defects
Yang et al. ** [72]	2022	USA	Cohort	2016–2019	National Vital Statistics System	Births without limb reduction defects	T1-T3	NS	NS	NS	NS	-	Maternal age Ethnicity Educational level Marital status Maternal BMI Eclampsia Gestational hypertension and diabetes Parity Infant sex Gestational age at delivery Total number of prenatal care visits	Limb reduction defects

* Adjusted odds ratios used where provided, otherwise crude odds ratios calculated from available data. ** Unable to identify total cases and control by smoking exposure alone as groups utilised in study were not independent. OR = Odds ratio. RR = Relative risk. NS = not specified. T1 = First Trimester. T1–T3 = Throughout Pregnancy.

**Table 3:** Quality assessment of included studies (Newcastle–Ottawa Scale for cohort and case-control studies).

Author	Year	Selection	Comparability	Outcome/Exposure	Quality
Kelsey et al. [48]	1978	★ ★ ★		★	Poor
Aro [43]	1983	★ ★ ★	★ ★	★ ★	Good
Aro et al. [54]	1984	★ ★	★ ★	★	Poor
Kricker et al. [35]	1986	★ ★ ★	★	★ ★	Fair
Shiono et al. [36]	1986	★ ★ ★	★ ★	★ ★ ★	Good
Kallen (A) [55]	1989	★ ★ ★	★	★ ★ ★	Fair
Van Den Eeden et al. [37]	1990	★ ★ ★	★	★ ★	Fair
Czeizel et al. (A) [56]	1994	★ ★ ★ ★		★	Poor
Wasserman et al. [57]	1996	★ ★ ★ ★		★ ★	Poor
Kallen (B) [58]	1997	★ ★ ★	★	★ ★	Fair
Carr [49]	1997	★ ★ ★		★ ★ ★	Poor
Martinez-Friar et al. (A) [47]	1999	★ ★ ★ ★	★	★	Poor
Martinez-Friar et al. (B) [47]	1999	★ ★ ★	★	★	Poor
Shaw et al. [59]	1999	★ ★ ★ ★		★ ★	Poor
Kallen (C) [32]	2000	★ ★ ★		★ ★	Poor
Honein et al. [29]	2001	★ ★ ★	★	★ ★	Fair
Woods and Raju [50]	2001	★ ★ ★ ★	★ ★	★ ★	Good
Shaw et al. [60]	2002	★ ★ ★ ★		★	Poor
Czeizel et al. (B) [62]	2004	★ ★ ★ ★		★	Poor
Carmichael et al. (A) [61]	2004	★ ★ ★ ★		★	Poor
Carmichael et al. (B) [63]	2006	★ ★ ★ ★		★	Poor
Carmichael et al. (C) [64]	2006	★ ★ ★ ★		★	Poor
Man and Chang [30]	2006	★ ★ ★	★	★ ★	Fair
Robitaille et al. [65]	2009	★ ★ ★ ★		★ ★	Poor
Werler et al. [38]	2009	★ ★ ★	★	★ ★	Fair
Srisukhumbowornchai et al. [66]	2012	★ ★ ★	★	★ ★	Fair
Caspers et al. [31]	2013	★ ★ ★ ★	★	★ ★	Fair
Leite et al. [51]	2014	★ ★ ★	★	★ ★	Fair
Kallen [34]	2014	★ ★ ★	★	★ ★	Fair
Pace et al. [67]	2018	★ ★ ★ ★		★ ★	Poor
Shi et al. [52]	2018	★ ★ ★	★	★ ★	Fair
Choi et al. [68]	2019	★ ★ ★ ★		★ ★	Poor
Klungsoyr et al. [69]	2019	★ ★ ★	★ ★	★ ★	Good
Perry et al. [33]	2019	★ ★ ★ ★	★ ★	★ ★	Good
Adrien et al. [70]	2020	★ ★ ★	★	★ ★	Fair
Materna-Kiryluk et al. [71]	2021	★ ★ ★		★ ★	Poor
Tsuchida et al. [53]	2021	★ ★ ★ ★	★ ★	★ ★	Good
Yang et al. [72]	2022	★ ★	★ ★	★ ★	Fair

Good quality: 3 or 4 stars (★) in selection domain AND 2 stars in comparability domain AND 2 or 3 stars in outcome/exposure domain. Fair quality: 2 stars in selection domain AND 1 or 2 stars in comparability domain AND 2 or 3 stars in outcome/exposure domain. Poor quality: 0 or 1 star in selection domain OR 0 stars in comparability domain OR 0 or 1 stars in outcome/exposure domain.

## Data Availability

Not applicable.
